# Practice by Companies

**Published:** 1900-07-07

**Authors:** 


### PRACTICE BY COMPANIES.

It is much to be hoped for many reasons that the clause of the Companies' Bill which prohibits the practice of medicine, surgery, midwifery, and dentistry by companies may escape that weeding out in Committee with which it is threatened. Notwithstanding the statement of the President of the Board of Trade to the effect that this clause is not germane to the Bill, we hold that in a Bill regulating the proceedings of public companies a clause limiting their sphere of action has a distinct *locus standi.* From a public point of view, especially bearing in mind the tendencies of the day which are all in the direction of the absorption of everything by stores and companies, it is of the greatest importance that those professions and those trades, the exercise of which has, for the protection of the public, been restricted by law to persons who have undergone a certain training, have passed certain examinations, and have been entered on certain registers, should not fall into the hands of public companies. The whole theory on which the training, examination, and registration of medical men and dentists—and, we may add, of pharmacists—is undertaken, and on which the law interferes with and regulates their work, is that the work is to be done personally by the individual, or under his immediate supervision; and it is a most serious matter that companies of absolutely unqualified persons should be allowed, as we fear is the case at the present time, to undertake work the doing of which by individuals has been strictly fenced round by Acts of Parliament, passed for the protection of the public against the pretences of unqualified persons.

